# Methyl 2-benzyl-5-[1-(4-methoxy­phen­yl)-4-oxo-3-phenyl­azetidin-2-yl]-4-nitro-3-phenyl­pyrrolidine-2-carboxyl­ate

**DOI:** 10.1107/S1600536808029875

**Published:** 2008-09-24

**Authors:** S. Sundaresan, P. Ramesh, N. Arumugam, R. Raghunathan, M. N. Ponnuswamy

**Affiliations:** a Centre of Advanced Study in Crystallography and Biophysics, University of Madras, Guindy Campus, Chennai 600 025, India; b Department of Physics, Presidency College (Autonomous), Chennai 600 005, India; c Department of Organic Chemistry, University of Madras, Guindy Campus, Chennai 600 025, India

## Abstract

In the title mol­ecule, C_35_H_33_N_3_O_6_, the pyrrolidine ring adopts a twist conformation. The mol­ecules are paired into centrosymmetric dimers by weak inter­molecular C—H⋯O hydrogen bonds. The dimers inter­act further again *via* C—H⋯O hydrogen bonds and N—H⋯O intramolecular interaction also stabilize the crystal packing.

## Related literature

For the pharmacological properties of β-lactam derivatives, see: Alcaide *et al.* (2000 ▶). For general background, see: Cremer & Pople (1975 ▶); Nardelli (1983 ▶); Beddoes *et al.* (1986 ▶).
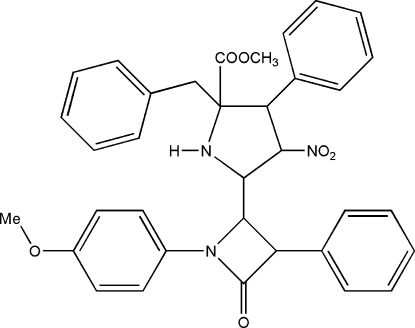


## Experimental

### 

#### Crystal data

C_35_H_33_N_3_O_6_
*M* *_r_* = 591.64
Triclinic, 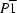

*a* = 10.1727 (2) Å
*b* = 10.4210 (2) Å
*c* = 15.1680 (3) Å
α = 91.833 (1)°
β = 106.154 (1)°
γ = 102.536 (1)°
*V* = 1500.31 (5) Å^3^
*Z* = 2
Mo *K*α radiation
μ = 0.09 mm^−1^
*T* = 293 (2) K
0.23 × 0.20 × 0.18 mm

#### Data collection

Bruker APEXII CCD area-detector diffractometer
Absorption correction: multi-scan (*SADABS*; Sheldrick, 2001 ▶) *T*_min_ = 0.978, *T*_max_ = 0.987
29479 measured reflections
5290 independent reflections
4498 reflections with *I* > 2σ(*I*)
*R*_int_ = 0.022

#### Refinement

*R*[*F* ^2^ > 2σ(*F* ^2^)] = 0.041
*wR*(*F* ^2^) = 0.112
*S* = 1.02
5290 reflections
400 parameters
1 restraint
H atoms treated by a mixture of independent and constrained refinement
Δρ_max_ = 0.28 e Å^−3^
Δρ_min_ = −0.28 e Å^−3^

### 

Data collection: *APEX2* (Bruker, 2004 ▶); cell refinement: *APEX2*; data reduction: *SAINT* (Bruker, 2004 ▶); program(s) used to solve structure: *SHELXS97* (Sheldrick, 2008 ▶); program(s) used to refine structure: *SHELXL97* (Sheldrick, 2008 ▶); molecular graphics: *ORTEP-3* (Farrugia, 1997 ▶); software used to prepare material for publication: *SHELXL97* and *PLATON* (Spek, 2003 ▶).

## Supplementary Material

Crystal structure: contains datablocks global, I. DOI: 10.1107/S1600536808029875/cv2439sup1.cif

Structure factors: contains datablocks I. DOI: 10.1107/S1600536808029875/cv2439Isup2.hkl

Additional supplementary materials:  crystallographic information; 3D view; checkCIF report

## Figures and Tables

**Table 1:** Hydrogen-bond geometry (Å, °)

*D*—H⋯*A*	*D*—H	H⋯*A*	*D*⋯*A*	*D*—H⋯*A*
N1—H1⋯O1	0.898 (19)	2.314 (18)	2.7378 (18)	108.7 (14)
C7—H7*B*⋯O3^i^	0.96	2.48	3.145 (2)	126
C14—H14⋯O5^ii^	0.93	2.60	3.359 (2)	139
C30—H30⋯O1^iii^	0.93	2.57	3.240 (2)	129

Symmetry codes: (i) 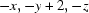; (ii) 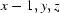; (iii) 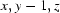.

**Fig. 1.**
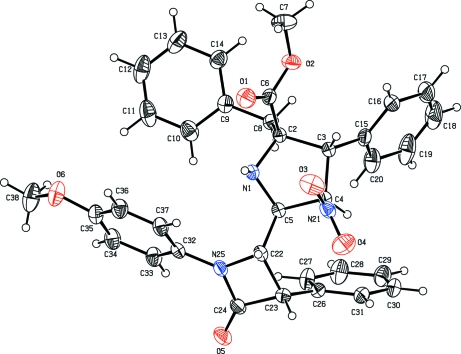
The molecular structure of (I) showing the atomic numbering and 50% probability displacement ellipsoids.

## supplementary crystallographic information

### Comment

An extensive use of common β-lactam antibiotics, such as penicillin and cephalosporins, in medicine has resulted in an increasing number of resistant bacteria through mutation and β-lactamase gene transfer. The importance and structural diversity of biologically active β-lactam antibiotics, the most widely employed family of antimicrobial agents led to the development of efficient approaches for the construction of appropriately substituted 2-azetidinones (Alcaide *et al.*, 2000). As a contribution to this field, we present here the crystal structure of the title compound, (I).

In (I) (Fig. 1), the pyrrolidine ring adopts a twist conformation. The puckering parameters (Cremer & Pople, 1975) and the asymmetry parameter (Nardelli, 1983) for this ring are q_2_ = 0.382 (2) Å, π = 340.9 (2)° and Δ2(C3) = 0.48 (14)°, respectively. The sum of angles at N1 of the pyrrolidine ring system [327.14 (12)°] is in accordance with *sp*^3^ hybridization (Beddoes *et al.*, 1986). The β-lactam ring is planar and the keto atom O5 deviates from this plane at 0.049 (1) Å.

The weak intermolecular C—H···O hydrogen bonds (Table 1) contribute to the crystal packing stability.

### Experimental

β-Lactam aldehyde (1.0 mol) was treated with phenyl alanine methyl ester hydrochloride in the presence of Et_3_N (2.5 mol) and anhydrous MgSO_4_ (2.0 g) in dry chloromethane (10 ml) at room temperature for 12 h to give the imine. The imine was washed with water and dried over Na_2_SO_4_. The solvent was evaporated under vacuum. The imine was then stirred with silver (I) acetate and nitrostyene (1.0 mol) in the presence of Et_3_N (1.2 mol) and molecular sieves in dry toluene (30 ml) again at room temperature for 12 h. The reaction mixture was filtered through a plug celite. The solvent was evaporated under reduced pressure and the residue was subjected to column chromatogaraphy on silicagel (100–200 mesh), with hexane and ethyl acetate (7:3) as eluent to give the product. The compound was recrystallized from ethyl acetate.

### Refinement

C-bound H atoms were geometrically positioned (C—H=0.93–0.98 Å) and refined as riding, with  *U*_iso_(H) = 1.5*U*_eq_(C) for methyl H 1.2*U*_eq_(C) for other H atoms. The H atom attached to N was located from difference Fourier map and isotropically refined with bond restraint N—H=0.90 (2) Å.

### Crystal data

**Table:**

C_35_H_33_N_3_O_6_	*Z* = 2
*M**_r_* = 591.64	*F*(000) = 624
Triclinic, *P*1	*D*_x_ = 1.310 Mg m^−^^3^
Hall symbol: -P 1	Mo *K*α radiation, λ = 0.71073 Å
*a* = 10.1727 (2) Å	Cell parameters from 4523 reflections
*b* = 10.4210 (2) Å	θ = 2.1–25.0°
*c* = 15.1680 (3) Å	µ = 0.09 mm^−^^1^
α = 91.833 (1)°	*T* = 293 K
β = 106.154 (1)°	Block, colourless
γ = 102.536 (1)°	0.23 × 0.20 × 0.18 mm
*V* = 1500.31 (5) Å^3^	

### Data collection

**Table:**

Bruker APEXII CCD area-detector diffractometer	5290 independent reflections
Radiation source: fine-focus sealed tube	4498 reflections with *I* > 2σ(*I*)
graphite	*R*_int_ = 0.022
ω and φ scans	θ_max_ = 25.0°, θ_min_ = 2.1°
Absorption correction: multi-scan (SADABS; Sheldrick, 2001)	*h* = −12→12
*T*_min_ = 0.978, *T*_max_ = 0.987	*k* = −12→12
29479 measured reflections	*l* = −18→18

### Refinement

**Table:**

Refinement on *F*^2^	Primary atom site location: structure-invariant direct methods
Least-squares matrix: full	Secondary atom site location: difference Fourier map
*R*[*F*^2^ > 2σ(*F*^2^)] = 0.041	Hydrogen site location: inferred from neighbouring sites
*wR*(*F*^2^) = 0.112	H atoms treated by a mixture of independent and constrained refinement
*S* = 1.02	*w* = 1/[σ^2^(*F*_o_^2^) + (0.0505*P*)^2^ + 0.5651*P*] where *P* = (*F*_o_^2^ + 2*F*_c_^2^)/3
5290 reflections	(Δ/σ)_max_ = 0.009
400 parameters	Δρ_max_ = 0.28 e Å^−^^3^
1 restraint	Δρ_min_ = −0.28 e Å^−^^3^

### Special details

**Table:**

Geometry. All e.s.d.'s (except the e.s.d. in the dihedral angle between two l.s. planes) are estimated using the full covariance matrix. The cell e.s.d.'s are taken into account individually in the estimation of e.s.d.'s in distances, angles and torsion angles; correlations between e.s.d.'s in cell parameters are only used when they are defined by crystal symmetry. An approximate (isotropic) treatment of cell e.s.d.'s is used for estimating e.s.d.'s involving l.s. planes.
Refinement. Refinement of *F*^2^ against ALL reflections. The weighted *R*-factor *wR* and goodness of fit *S* are based on *F*^2^, conventional *R*-factors *R* are based on *F*, with *F* set to zero for negative *F*^2^. The threshold expression of *F*^2^ > σ(*F*^2^) is used only for calculating *R*-factors(gt) *etc*. and is not relevant to the choice of reflections for refinement. *R*-factors based on *F*^2^ are statistically about twice as large as those based on *F*, and *R*- factors based on ALL data will be even larger.

### Fractional atomic coordinates and isotropic or equivalent isotropic displacement parameters (Å^2^)

**Table:**

	*x*	*y*	*z*	*U*_iso_*/*U*_eq_	
O1	0.23296 (14)	1.11254 (12)	0.12037 (10)	0.0626 (4)	
O2	0.05010 (12)	0.97536 (12)	0.14730 (9)	0.0516 (3)	
O3	0.34127 (15)	0.89947 (15)	−0.01081 (9)	0.0693 (4)	
O4	0.43868 (18)	0.73840 (17)	−0.01654 (10)	0.0826 (5)	
O5	0.89861 (13)	0.81334 (13)	0.32648 (10)	0.0665 (4)	
O6	0.83342 (17)	1.40874 (13)	0.47301 (9)	0.0727 (4)	
N1	0.41420 (13)	0.96169 (12)	0.20111 (9)	0.0360 (3)	
H1	0.4247 (19)	1.0180 (18)	0.1586 (13)	0.050 (5)*	
C2	0.26396 (15)	0.91317 (14)	0.19096 (10)	0.0344 (3)	
C3	0.21203 (15)	0.78623 (14)	0.11656 (10)	0.0351 (3)	
H3	0.1651	0.8153	0.0577	0.042*	
C4	0.34889 (15)	0.75799 (14)	0.10697 (10)	0.0352 (3)	
H4	0.3481	0.6642	0.1114	0.042*	
C5	0.46471 (14)	0.84462 (14)	0.18892 (9)	0.0333 (3)	
H5	0.4641	0.7975	0.2437	0.040*	
C6	0.18470 (16)	1.01459 (14)	0.14978 (10)	0.0393 (3)	
C7	−0.0397 (2)	1.0629 (2)	0.11332 (17)	0.0759 (6)	
H7A	−0.0355	1.1242	0.1632	0.114*	
H7B	−0.1348	1.0125	0.0873	0.114*	
H7C	−0.0087	1.1105	0.0668	0.114*	
C8	0.24250 (17)	0.88720 (15)	0.28635 (10)	0.0390 (3)	
H8A	0.1451	0.8427	0.2782	0.047*	
H8B	0.3011	0.8292	0.3153	0.047*	
C9	0.27898 (17)	1.01341 (15)	0.34880 (10)	0.0421 (4)	
C10	0.4168 (2)	1.0744 (2)	0.39661 (12)	0.0592 (5)	
H10	0.4893	1.0358	0.3923	0.071*	
C11	0.4474 (3)	1.1928 (2)	0.45082 (14)	0.0774 (7)	
H11	0.5404	1.2330	0.4827	0.093*	
C12	0.3418 (3)	1.2512 (2)	0.45787 (14)	0.0780 (7)	
H12	0.3632	1.3313	0.4936	0.094*	
C13	0.2051 (3)	1.1913 (2)	0.41225 (15)	0.0690 (6)	
H13	0.1329	1.2300	0.4173	0.083*	
C14	0.1743 (2)	1.07297 (17)	0.35862 (13)	0.0533 (4)	
H14	0.0808	1.0323	0.3283	0.064*	
C15	0.11103 (17)	0.66599 (15)	0.13188 (12)	0.0450 (4)	
C16	−0.02995 (19)	0.64235 (19)	0.08179 (17)	0.0655 (6)	
H16	−0.0610	0.7028	0.0421	0.079*	
C17	−0.1244 (3)	0.5302 (3)	0.0903 (2)	0.0917 (9)	
H17	−0.2185	0.5149	0.0558	0.110*	
C18	−0.0802 (3)	0.4412 (3)	0.1494 (2)	0.0987 (10)	
H18	−0.1445	0.3664	0.1559	0.118*	
C19	0.0593 (3)	0.4625 (2)	0.19912 (17)	0.0898 (9)	
H19	0.0896	0.4015	0.2386	0.108*	
C20	0.15546 (12)	0.57555 (12)	0.19041 (7)	0.0653 (5)	
H20	0.2498	0.5899	0.2242	0.078*	
N21	0.37621 (12)	0.80108 (12)	0.01871 (7)	0.0463 (3)	
C22	0.61299 (15)	0.86856 (14)	0.18124 (10)	0.0358 (3)	
H22	0.6225	0.9148	0.1273	0.043*	
C23	0.68163 (16)	0.74593 (15)	0.18950 (11)	0.0409 (4)	
H23	0.7200	0.7330	0.1382	0.049*	
C24	0.79266 (16)	0.82884 (16)	0.27273 (12)	0.0453 (4)	
N25	0.72602 (13)	0.92975 (12)	0.26579 (9)	0.0400 (3)	
C26	0.60056 (16)	0.61819 (15)	0.21114 (11)	0.0398 (3)	
C27	0.5883 (2)	0.60247 (17)	0.29894 (12)	0.0544 (4)	
H27	0.6350	0.6700	0.3460	0.065*	
C28	0.5075 (2)	0.48762 (19)	0.31760 (14)	0.0665 (6)	
H28	0.4998	0.4789	0.3769	0.080*	
C29	0.4386 (2)	0.38636 (18)	0.24931 (14)	0.0612 (5)	
H29	0.3833	0.3097	0.2619	0.073*	
C30	0.45205 (19)	0.39919 (17)	0.16239 (13)	0.0543 (4)	
H30	0.4066	0.3306	0.1159	0.065*	
C31	0.53301 (17)	0.51375 (16)	0.14369 (12)	0.0468 (4)	
H31	0.5423	0.5208	0.0846	0.056*	
C32	0.75777 (15)	1.05282 (15)	0.31897 (10)	0.0387 (3)	
C33	0.81676 (18)	1.06067 (17)	0.41328 (11)	0.0492 (4)	
H33	0.8368	0.9859	0.4408	0.059*	
C34	0.84635 (19)	1.17897 (18)	0.46717 (12)	0.0535 (4)	
H34	0.8872	1.1841	0.5306	0.064*	
C35	0.81481 (19)	1.28903 (17)	0.42629 (12)	0.0499 (4)	
C36	0.75824 (19)	1.28152 (17)	0.33134 (12)	0.0519 (4)	
H36	0.7393	1.3564	0.3036	0.062*	
C37	0.73000 (17)	1.16421 (16)	0.27817 (11)	0.0449 (4)	
H37	0.6921	1.1598	0.2145	0.054*	
C38	0.8872 (3)	1.4202 (2)	0.57069 (15)	0.0889 (8)	
H38A	0.8229	1.3612	0.5954	0.133*	
H38B	0.8980	1.5093	0.5949	0.133*	
H38C	0.9769	1.3976	0.5878	0.133*	

### Atomic displacement parameters (Å^2^)

**Table:**

	*U*^11^	*U*^22^	*U*^33^	*U*^12^	*U*^13^	*U*^23^
O1	0.0623 (8)	0.0387 (7)	0.0866 (10)	0.0095 (6)	0.0213 (7)	0.0223 (6)
O2	0.0399 (6)	0.0535 (7)	0.0648 (8)	0.0186 (5)	0.0139 (5)	0.0148 (6)
O3	0.0656 (9)	0.0890 (10)	0.0612 (8)	0.0237 (8)	0.0238 (7)	0.0355 (8)
O4	0.0925 (11)	0.1026 (12)	0.0673 (9)	0.0283 (10)	0.0449 (9)	−0.0130 (8)
O5	0.0401 (7)	0.0584 (8)	0.0846 (10)	0.0125 (6)	−0.0084 (6)	0.0024 (7)
O6	0.1005 (11)	0.0491 (8)	0.0573 (8)	0.0088 (7)	0.0136 (7)	−0.0094 (6)
N1	0.0327 (7)	0.0317 (7)	0.0411 (7)	0.0024 (5)	0.0109 (5)	0.0018 (5)
C2	0.0316 (7)	0.0306 (7)	0.0388 (8)	0.0036 (6)	0.0095 (6)	0.0020 (6)
C3	0.0315 (7)	0.0329 (8)	0.0378 (8)	0.0040 (6)	0.0083 (6)	0.0006 (6)
C4	0.0326 (8)	0.0333 (8)	0.0366 (8)	0.0046 (6)	0.0079 (6)	0.0009 (6)
C5	0.0318 (7)	0.0334 (7)	0.0320 (7)	0.0040 (6)	0.0078 (6)	0.0028 (6)
C6	0.0422 (9)	0.0321 (8)	0.0410 (8)	0.0069 (7)	0.0099 (7)	0.0007 (6)
C7	0.0667 (13)	0.0884 (16)	0.0871 (15)	0.0484 (12)	0.0209 (11)	0.0243 (12)
C8	0.0400 (8)	0.0355 (8)	0.0433 (8)	0.0080 (6)	0.0155 (7)	0.0062 (6)
C9	0.0494 (9)	0.0405 (8)	0.0364 (8)	0.0042 (7)	0.0174 (7)	0.0052 (6)
C10	0.0550 (11)	0.0741 (13)	0.0402 (9)	0.0027 (9)	0.0111 (8)	−0.0032 (8)
C11	0.0784 (15)	0.0843 (16)	0.0468 (11)	−0.0218 (13)	0.0156 (10)	−0.0148 (10)
C12	0.121 (2)	0.0534 (12)	0.0555 (12)	−0.0122 (13)	0.0460 (13)	−0.0123 (9)
C13	0.1007 (17)	0.0483 (11)	0.0701 (13)	0.0140 (11)	0.0481 (13)	−0.0011 (9)
C14	0.0611 (11)	0.0469 (10)	0.0563 (10)	0.0085 (8)	0.0278 (9)	0.0004 (8)
C15	0.0461 (9)	0.0346 (8)	0.0533 (9)	−0.0028 (7)	0.0239 (8)	−0.0086 (7)
C16	0.0402 (10)	0.0508 (11)	0.1018 (16)	−0.0018 (8)	0.0269 (10)	−0.0182 (10)
C17	0.0582 (14)	0.0649 (15)	0.147 (3)	−0.0187 (12)	0.0532 (15)	−0.0343 (16)
C18	0.112 (2)	0.0620 (15)	0.121 (2)	−0.0379 (15)	0.080 (2)	−0.0239 (15)
C19	0.143 (3)	0.0435 (11)	0.0733 (15)	−0.0174 (12)	0.0472 (16)	−0.0008 (10)
C20	0.0855 (15)	0.0458 (10)	0.0552 (11)	−0.0057 (9)	0.0221 (10)	0.0024 (8)
N21	0.0357 (7)	0.0606 (9)	0.0356 (7)	0.0024 (6)	0.0071 (6)	−0.0049 (6)
C22	0.0321 (8)	0.0377 (8)	0.0335 (7)	0.0021 (6)	0.0081 (6)	0.0027 (6)
C23	0.0353 (8)	0.0430 (9)	0.0446 (8)	0.0088 (7)	0.0128 (7)	−0.0002 (7)
C24	0.0314 (8)	0.0437 (9)	0.0556 (10)	0.0053 (7)	0.0070 (7)	0.0049 (7)
N25	0.0313 (6)	0.0392 (7)	0.0417 (7)	0.0039 (5)	0.0021 (5)	0.0000 (5)
C26	0.0378 (8)	0.0364 (8)	0.0442 (8)	0.0113 (6)	0.0089 (6)	−0.0004 (6)
C27	0.0722 (12)	0.0402 (9)	0.0420 (9)	0.0042 (8)	0.0101 (8)	−0.0016 (7)
C28	0.0963 (16)	0.0480 (11)	0.0496 (10)	0.0039 (10)	0.0220 (10)	0.0089 (8)
C29	0.0716 (13)	0.0393 (10)	0.0695 (13)	0.0046 (9)	0.0220 (10)	0.0065 (8)
C30	0.0542 (11)	0.0395 (9)	0.0630 (11)	0.0047 (8)	0.0139 (9)	−0.0107 (8)
C31	0.0459 (9)	0.0468 (9)	0.0470 (9)	0.0100 (7)	0.0147 (7)	−0.0069 (7)
C32	0.0301 (7)	0.0392 (8)	0.0411 (8)	0.0009 (6)	0.0073 (6)	0.0020 (6)
C33	0.0527 (10)	0.0442 (9)	0.0423 (9)	0.0062 (8)	0.0038 (7)	0.0067 (7)
C34	0.0576 (11)	0.0546 (10)	0.0370 (9)	0.0031 (8)	0.0038 (7)	0.0007 (7)
C35	0.0508 (10)	0.0430 (9)	0.0480 (9)	−0.0006 (8)	0.0117 (8)	−0.0037 (7)
C36	0.0572 (11)	0.0418 (9)	0.0508 (10)	0.0072 (8)	0.0096 (8)	0.0077 (7)
C37	0.0459 (9)	0.0437 (9)	0.0373 (8)	0.0029 (7)	0.0056 (7)	0.0050 (7)
C38	0.131 (2)	0.0645 (14)	0.0560 (13)	−0.0010 (14)	0.0233 (13)	−0.0162 (10)

### Geometric parameters (Å, °)

**Table:**

O1—C6	1.1896 (19)	C16—C17	1.377 (3)
O2—C6	1.3307 (19)	C16—H16	0.9300
O2—C7	1.436 (2)	C17—C18	1.370 (4)
O3—N21	1.2154 (17)	C17—H17	0.9300
O4—N21	1.2098 (18)	C18—C19	1.375 (4)
O5—C24	1.203 (2)	C18—H18	0.9300
O6—C35	1.364 (2)	C19—C20	1.394 (2)
O6—C38	1.421 (3)	C19—H19	0.9300
N1—C5	1.4482 (19)	C20—H20	0.9300
N1—C2	1.4642 (18)	C22—N25	1.4773 (18)
N1—H1	0.898 (19)	C22—C23	1.577 (2)
C2—C6	1.513 (2)	C22—H22	0.9800
C2—C8	1.546 (2)	C23—C26	1.504 (2)
C2—C3	1.6036 (19)	C23—C24	1.526 (2)
C3—C15	1.507 (2)	C23—H23	0.9800
C3—C4	1.528 (2)	C24—N25	1.361 (2)
C3—H3	0.9800	N25—C32	1.417 (2)
C4—N21	1.5040 (18)	C26—C27	1.383 (2)
C4—C5	1.5491 (19)	C26—C31	1.384 (2)
C4—H4	0.9800	C27—C28	1.383 (3)
C5—C22	1.512 (2)	C27—H27	0.9300
C5—H5	0.9800	C28—C29	1.372 (3)
C7—H7A	0.9600	C28—H28	0.9300
C7—H7B	0.9600	C29—C30	1.370 (3)
C7—H7C	0.9600	C29—H29	0.9300
C8—C9	1.509 (2)	C30—C31	1.382 (2)
C8—H8A	0.9700	C30—H30	0.9300
C8—H8B	0.9700	C31—H31	0.9300
C9—C14	1.382 (2)	C32—C37	1.379 (2)
C9—C10	1.383 (2)	C32—C33	1.381 (2)
C10—C11	1.386 (3)	C33—C34	1.384 (2)
C10—H10	0.9300	C33—H33	0.9300
C11—C12	1.371 (4)	C34—C35	1.378 (3)
C11—H11	0.9300	C34—H34	0.9300
C12—C13	1.365 (3)	C35—C36	1.387 (2)
C12—H12	0.9300	C36—C37	1.372 (2)
C13—C14	1.381 (3)	C36—H36	0.9300
C13—H13	0.9300	C37—H37	0.9300
C14—H14	0.9300	C38—H38A	0.9600
C15—C20	1.377 (2)	C38—H38B	0.9600
C15—C16	1.387 (3)	C38—H38C	0.9600
			
C6—O2—C7	117.27 (15)	C17—C18—H18	120.0
C35—O6—C38	117.72 (16)	C19—C18—H18	120.0
C5—N1—C2	105.14 (11)	C18—C19—C20	120.0 (2)
C5—N1—H1	112.7 (12)	C18—C19—H19	120.0
C2—N1—H1	109.2 (12)	C20—C19—H19	120.0
N1—C2—C6	109.82 (12)	C15—C20—C19	120.13 (17)
N1—C2—C8	109.67 (12)	C15—C20—H20	119.9
C6—C2—C8	109.45 (12)	C19—C20—H20	119.9
N1—C2—C3	105.46 (11)	O4—N21—O3	123.59 (14)
C6—C2—C3	107.03 (11)	O4—N21—C4	117.29 (14)
C8—C2—C3	115.26 (11)	O3—N21—C4	119.01 (12)
C15—C3—C4	113.48 (12)	N25—C22—C5	115.57 (12)
C15—C3—C2	119.09 (12)	N25—C22—C23	86.76 (11)
C4—C3—C2	103.76 (11)	C5—C22—C23	116.52 (12)
C15—C3—H3	106.6	N25—C22—H22	111.9
C4—C3—H3	106.6	C5—C22—H22	111.9
C2—C3—H3	106.6	C23—C22—H22	111.9
N21—C4—C3	111.73 (12)	C26—C23—C24	114.59 (13)
N21—C4—C5	108.44 (11)	C26—C23—C22	118.28 (12)
C3—C4—C5	104.15 (11)	C24—C23—C22	84.95 (11)
N21—C4—H4	110.8	C26—C23—H23	112.1
C3—C4—H4	110.8	C24—C23—H23	112.1
C5—C4—H4	110.8	C22—C23—H23	112.1
N1—C5—C22	115.85 (12)	O5—C24—N25	132.13 (16)
N1—C5—C4	104.92 (11)	O5—C24—C23	134.79 (16)
C22—C5—C4	115.49 (12)	N25—C24—C23	93.07 (12)
N1—C5—H5	106.6	C24—N25—C32	132.30 (13)
C22—C5—H5	106.6	C24—N25—C22	95.07 (12)
C4—C5—H5	106.6	C32—N25—C22	132.58 (12)
O1—C6—O2	124.43 (15)	C27—C26—C31	117.78 (15)
O1—C6—C2	125.63 (15)	C27—C26—C23	120.89 (14)
O2—C6—C2	109.88 (12)	C31—C26—C23	121.31 (14)
O2—C7—H7A	109.5	C28—C27—C26	120.84 (16)
O2—C7—H7B	109.5	C28—C27—H27	119.6
H7A—C7—H7B	109.5	C26—C27—H27	119.6
O2—C7—H7C	109.5	C29—C28—C27	120.53 (18)
H7A—C7—H7C	109.5	C29—C28—H28	119.7
H7B—C7—H7C	109.5	C27—C28—H28	119.7
C9—C8—C2	111.98 (12)	C30—C29—C28	119.38 (17)
C9—C8—H8A	109.2	C30—C29—H29	120.3
C2—C8—H8A	109.2	C28—C29—H29	120.3
C9—C8—H8B	109.2	C29—C30—C31	120.16 (16)
C2—C8—H8B	109.2	C29—C30—H30	119.9
H8A—C8—H8B	107.9	C31—C30—H30	119.9
C14—C9—C10	117.83 (16)	C30—C31—C26	121.27 (16)
C14—C9—C8	120.43 (15)	C30—C31—H31	119.4
C10—C9—C8	121.74 (16)	C26—C31—H31	119.4
C9—C10—C11	120.4 (2)	C37—C32—C33	119.64 (15)
C9—C10—H10	119.8	C37—C32—N25	121.12 (14)
C11—C10—H10	119.8	C33—C32—N25	119.24 (14)
C12—C11—C10	120.6 (2)	C32—C33—C34	120.50 (16)
C12—C11—H11	119.7	C32—C33—H33	119.8
C10—C11—H11	119.7	C34—C33—H33	119.8
C13—C12—C11	119.76 (19)	C35—C34—C33	119.57 (16)
C13—C12—H12	120.1	C35—C34—H34	120.2
C11—C12—H12	120.1	C33—C34—H34	120.2
C12—C13—C14	119.7 (2)	O6—C35—C34	124.53 (16)
C12—C13—H13	120.1	O6—C35—C36	115.68 (16)
C14—C13—H13	120.1	C34—C35—C36	119.79 (16)
C13—C14—C9	121.67 (19)	C37—C36—C35	120.38 (16)
C13—C14—H14	119.2	C37—C36—H36	119.8
C9—C14—H14	119.2	C35—C36—H36	119.8
C20—C15—C16	119.00 (15)	C36—C37—C32	120.09 (15)
C20—C15—C3	122.10 (14)	C36—C37—H37	120.0
C16—C15—C3	118.82 (17)	C32—C37—H37	120.0
C17—C16—C15	120.7 (2)	O6—C38—H38A	109.5
C17—C16—H16	119.7	O6—C38—H38B	109.5
C15—C16—H16	119.7	H38A—C38—H38B	109.5
C18—C17—C16	120.2 (3)	O6—C38—H38C	109.5
C18—C17—H17	119.9	H38A—C38—H38C	109.5
C16—C17—H17	119.9	H38B—C38—H38C	109.5
C17—C18—C19	120.0 (2)		
			
C5—N1—C2—C6	−148.46 (12)	C3—C4—N21—O4	−148.71 (14)
C5—N1—C2—C8	91.23 (13)	C5—C4—N21—O4	97.06 (16)
C5—N1—C2—C3	−33.45 (14)	C3—C4—N21—O3	34.86 (17)
N1—C2—C3—C15	139.14 (14)	C5—C4—N21—O3	−79.36 (16)
C6—C2—C3—C15	−103.94 (15)	N1—C5—C22—N25	67.52 (16)
C8—C2—C3—C15	18.03 (19)	C4—C5—C22—N25	−169.25 (12)
N1—C2—C3—C4	11.85 (14)	N1—C5—C22—C23	167.24 (12)
C6—C2—C3—C4	128.77 (12)	C4—C5—C22—C23	−69.53 (16)
C8—C2—C3—C4	−109.26 (13)	N25—C22—C23—C26	112.64 (14)
C15—C3—C4—N21	124.95 (13)	C5—C22—C23—C26	−4.42 (19)
C2—C3—C4—N21	−104.33 (12)	N25—C22—C23—C24	−2.62 (11)
C15—C3—C4—C5	−118.20 (13)	C5—C22—C23—C24	−119.68 (13)
C2—C3—C4—C5	12.52 (14)	C26—C23—C24—O5	62.9 (3)
C2—N1—C5—C22	170.75 (12)	C22—C23—C24—O5	−178.2 (2)
C2—N1—C5—C4	42.13 (14)	C26—C23—C24—N25	−116.01 (14)
N21—C4—C5—N1	85.54 (13)	C22—C23—C24—N25	2.85 (12)
C3—C4—C5—N1	−33.58 (14)	O5—C24—N25—C32	0.4 (3)
N21—C4—C5—C22	−43.29 (16)	C23—C24—N25—C32	179.33 (15)
C3—C4—C5—C22	−162.40 (12)	O5—C24—N25—C22	178.0 (2)
C7—O2—C6—O1	−5.1 (3)	C23—C24—N25—C22	−3.04 (12)
C7—O2—C6—C2	177.64 (15)	C5—C22—N25—C24	120.89 (14)
N1—C2—C6—O1	7.7 (2)	C23—C22—N25—C24	2.94 (12)
C8—C2—C6—O1	128.15 (17)	C5—C22—N25—C32	−61.5 (2)
C3—C2—C6—O1	−106.30 (18)	C23—C22—N25—C32	−179.44 (16)
N1—C2—C6—O2	−175.08 (12)	C24—C23—C26—C27	23.7 (2)
C8—C2—C6—O2	−54.63 (16)	C22—C23—C26—C27	−74.1 (2)
C3—C2—C6—O2	70.92 (15)	C24—C23—C26—C31	−157.88 (15)
N1—C2—C8—C9	67.48 (16)	C22—C23—C26—C31	104.29 (17)
C6—C2—C8—C9	−53.05 (16)	C31—C26—C27—C28	−2.0 (3)
C3—C2—C8—C9	−173.72 (12)	C23—C26—C27—C28	176.51 (17)
C2—C8—C9—C14	98.48 (17)	C26—C27—C28—C29	0.5 (3)
C2—C8—C9—C10	−80.41 (19)	C27—C28—C29—C30	0.9 (3)
C14—C9—C10—C11	−1.3 (3)	C28—C29—C30—C31	−0.7 (3)
C8—C9—C10—C11	177.64 (16)	C29—C30—C31—C26	−0.8 (3)
C9—C10—C11—C12	0.0 (3)	C27—C26—C31—C30	2.1 (3)
C10—C11—C12—C13	1.0 (3)	C23—C26—C31—C30	−176.33 (15)
C11—C12—C13—C14	−0.6 (3)	C24—N25—C32—C37	143.82 (17)
C12—C13—C14—C9	−0.7 (3)	C22—N25—C32—C37	−33.0 (2)
C10—C9—C14—C13	1.7 (3)	C24—N25—C32—C33	−36.6 (2)
C8—C9—C14—C13	−177.26 (16)	C22—N25—C32—C33	146.66 (16)
C4—C3—C15—C20	41.98 (19)	C37—C32—C33—C34	0.9 (3)
C2—C3—C15—C20	−80.61 (18)	N25—C32—C33—C34	−178.74 (15)
C4—C3—C15—C16	−134.62 (15)	C32—C33—C34—C35	0.9 (3)
C2—C3—C15—C16	102.79 (17)	C38—O6—C35—C34	−0.9 (3)
C20—C15—C16—C17	0.0 (3)	C38—O6—C35—C36	178.2 (2)
C3—C15—C16—C17	176.73 (17)	C33—C34—C35—O6	176.89 (17)
C15—C16—C17—C18	0.8 (3)	C33—C34—C35—C36	−2.2 (3)
C16—C17—C18—C19	−1.2 (4)	O6—C35—C36—C37	−177.39 (16)
C17—C18—C19—C20	0.9 (4)	C34—C35—C36—C37	1.8 (3)
C16—C15—C20—C19	−0.3 (2)	C35—C36—C37—C32	0.0 (3)
C3—C15—C20—C19	−176.93 (16)	C33—C32—C37—C36	−1.3 (2)
C18—C19—C20—C15	−0.1 (3)	N25—C32—C37—C36	178.29 (15)

### Hydrogen-bond geometry (Å, °)

**Table:**

*D*—H···*A*	*D*—H	H···*A*	*D*···*A*	*D*—H···*A*
N1—H1···O1	0.898 (19)	2.314 (18)	2.7378 (18)	108.7 (14)
C7—H7B···O3^i^	0.96	2.48	3.145 (2)	126.
C14—H14···O5^ii^	0.93	2.60	3.359 (2)	139.
C30—H30···O1^iii^	0.93	2.57	3.240 (2)	129.

Symmetry codes:  (i) −*x*, −*y*+2, −*z*; (ii) *x*−1, *y*, *z*; (iii) *x*, *y*−1, *z*.
